# EXTENDING GERONTOLOGICAL KNOWLEDGE AND THEORY THROUGH ETHNOGRAPHIC CASE STUDY METHODS

**DOI:** 10.1093/geroni/igae098.1971

**Published:** 2024-12-31

**Authors:** Amanda Grenier

**Affiliations:** University of Toronto, Toronto, Ontario, Canada

## Abstract

Experiences of older people deemed to be ‘at risk or ‘in need’ of intervention are often viewed through professional classifications with corresponding ‘objective’ indices. Concepts such as frailty and mobility, which have come to draw international policy attention, and arguably shape understandings of aging, are also produced within disciplinary knowledge(s) and powerful practices that prioritize particular components over others. As critical perspectives have revealed, such processes can marginalize groups of older people, resulting in research that articulates counter positions based on disjuncture between classifications, responses, and experience. Yet, operating in the inter-disciplinary, bio-medical/technocratic, and applied contexts of gerontology, research findings themselves become sets of knowledge and discourse which are understood as binaries, with the associated sociological theoretical perspectives often marginalized in the process. This paper suggests that ethnographic case study methods can be used to better understand the social complexities of aging, including the framing and interpretation of experience(s), the everyday contexts where older people negotiate and enact relationships and lives over time, and the ways in which evidence is used to design and respond to (or deny) older people’s needs. It takes a critical position focused on the production of knowledge and the experiences of older people in the context of social, cultural, and political relations, arguing for methods which render visible the complex realities of aging that are constructed, experienced and lived through, in space and time. It outlines the ethnographic case study as one potential method to carry out this work, presenting examples on frailty and (im)mobility.

